# *In Vitro* Whole Blood Clot Lysis for Fibrinolytic Activity Study Using D-Dimer and Confocal Microscopy

**DOI:** 10.1155/2014/814684

**Published:** 2014-02-11

**Authors:** Abuzar Elnager, Wan Zaidah Abdullah, Rosline Hassan, Zamzuri Idris, Nadiah Wan Arfah, S. A. Sulaiman, Zulkifli Mustafa

**Affiliations:** ^1^Department of Haematology, School of Medical Sciences, Universiti Sains Malaysia, Health Campus, 16150 Kubang Kerian, Kelantan, Malaysia; ^2^Department of Neurosciences, School of Medical Sciences, Universiti Sains Malaysia, Health Campus, 16150 Kubang Kerian, Kelantan, Malaysia; ^3^Unit of Biostatistics and Research Methodology, School of Medical Sciences, Universiti Sains Malaysia, Health Campus, 16150 Kubang Kerian, Kelantan, Malaysia; ^4^Department of Pharmacology, School of Medical Sciences, Universiti Sains Malaysia, Health Campus, 16150 Kubang Kerian, Kelantan, Malaysia

## Abstract

This study aimed to evaluate *in vitro* whole blood (WB) clot lysis method for the assessment of fibrinolytic activity. Standardized unresected (uncut) retracted WB clot was incubated in pool platelet poor plasma (PPP) for varying incubation times and in streptokinase (SK) at different concentrations. The fibrinolytic activity was assessed by D-dimer (DD), confocal microscopy, and clot weight. DD was measured photometrically by immunoturbidimetric method. There was a significant difference in mean DD levels according to SK concentrations (*P* = 0.007). The mean DD ± SD according to the SK concentrations of 5, 30, 50, and 100 IU/mL was: 0.69 ± 0.12, 0.78 ± 0.14, 1.04 ± 0.14 and 2.40 ± 1.09 **μ**g/mL. There were no significant changes of clot weight at different SK concentrations. Gradual loss and increased branching of fibrin in both PPP and SK were observed. Quantitation of DD and morphology of fibrin loss as observed by the imaging features are in keeping with fibrinolytic activity. Combination of DD levels and confocal microscopic features was successfully applied to evaluate the *in vitro* WB clot lysis method described here.

## 1. Introduction

Fibrinolysis is a mechanism of fibrin breakdown in blood clot occurring *in vivo* or *in vitro* through a physiological process or by therapeutic induction. Naturally, fibrinolysis is a result of plasmin serine protease pathway activation. Fibrinolytic agent induces enzymatic activation of plasminogen to plasmin which cleaves the fibrin molecules [1–4].

The quantity of DD reflects intravascular levels of fibrin turnover, without significant interference from fibrinogen or soluble fibrin degradation products, indicating that both thrombin generation and plasmin generation have occurred [5].

Prasad et al. in 2006 developed an *in vitro *clot lytic model using streptokinase (SK) based on the percentage of clot lysis and found that increased percentage of lysis occurred according to the concentrations of SK [6]. Other *in vitro* studies investigated the association of blood plasma flow characteristics using recombinant tissue-plasminogen activator (rt-PA) or SK on the degradation of retracted (aged) and nonretracted (fresh) WB clot under a special perfusion system. They concluded that the size of clot fragments and the frequency of their removal increase with direct flow of plasma or flow velocity [4, 7, 8].

A few other *in vitro* studies of WB clot lysis reported the use of ^125^I-fibrin labeled rt-PA, recombinant two chain urokinase-type plasminogen activator (rtcu-PA), and SK. They found that the rate of release of ^125^I-fibrin was an indicator of clot lysis [9–11]. Other researchers used the rate of hemoglobin release as determined by cyanomethemoglobin technique as indicator of clot dissolution which was expressed as percentage of lysis [12]. On the other hand, a few studies reported on plasma clot containing washed red blood cells (RBCs) with different concentrations to determine the effects of RBCs on fibrin clot structure as assisted by confocal microscopy. They noted that different concentrations had an effect on fibrin arrangement in a clot structure [13, 14].

There are very few reports that have highlighted the *in vitro* procedure for WB clot lysis by using DD and structural evaluation for fibrinolytic activity assessment. It is thought that WB clot structure (compared to fibrin clot) has an advantage to reflect the closest similarity between *in vitro *and *in vivo *process of lytic activity with the exception of the vascular milieu. WB clot mimics the *in vivo* clot which contains all the components of blood such as RBCs, platelets, and white blood cells.

The aim of the present study was to validate and standardize an *in vitro* WB clot method for fibrinolysis related studies. The validation method used SK as an inducer of fibrinolysis and PPP as controls for time factor. The fibrinolytic activity was assessed quantitatively by measurement of DD concentrations (a specific fibrinolytic marker) and clot weight and qualitatively by WB clot morphology using confocal microscopy. These tools were used to validate the procedure for *in vitro* WB clot lysis method for fibrinolytic activity which could be performed in a clinical laboratory. With the advances of laboratory automation (measuring DD, etc.), more works for research purposes could be applied as a preliminary study. So far no report had combined the structural and molecular measurement of fibrinolysis from a WB clot for the *in vitro* fibrinolytic activity study.

## 2. Methodology

### 2.1. *In Vitro* WB Clot Procedure for Fibrinolytic Activity Studies

#### 2.1.1. Preparation of Normal Pool Plasma

Following local Institutional Ethical Board approval of the protocol, an informed consent was obtained and then human PPP was prepared and processed strictly using O blood group as a source of plasma by collecting about 9 mL of WB from each donor into two trisodium citrate tubes (4.5 mL each). Blood free from HIV and hepatitis B antigen (Ag) was drawn from volunteers using evacuated system with multisample needle green sterile 21GX1(1/2). Immediately after collection, samples were spun using a centrifuge (Eppendorf, 5810 R, Germany) at 1500 g for 15 minutes at room temperature and then the supernatant was spun down at 1200 g for 15 min. The procedure was carried out according to the Clinical Laboratory Standardization Institute (CLSI) guideline for coagulation tests. Next, the PPP of all donors was pooled and the coagulation profile including fibrinolytic markers was measured to obtain standardized pool PPP especially tested with prothrombin time, activated partial prothrombin time, fibrinogen, and DD using STA compact, and ACL Elite-Procoagulation Analyzers (Diagnostic STAGO, France, and Instrumentation Laboratory, Italy, resp.). The standardized pool PPP was aliquot into small volume (1 mL) in cryogenic vial and stored at −80°C for further study.

#### 2.1.2. WB Clot Preparation

Venous blood (4.5 mL) was drawn from one healthy volunteer from blood group O donor so as to maintain the consistency of the results. The blood was then transferred into three preweighed sterile siliconized glass tubes 12 × 75 mm without anticoagulant. It was first allowed to clot at room temperature for approximately 10 min. The tube was covered by parafilm to prevent contamination and haemolysis from water when it was incubated in water bath. It was then incubated at 37°C in controlled temperature water bath (Grant SUB6 England) for 3 hrs to ensure complete clot retraction. After the WB clot completely retracted from the edges of the glass tube, the serum was removed using Pasteur pipette. The tubes were dried by using filter paper and each tube with clot was again weighed to determine the clot weight (clot weight = weight of clot containing tube − weight of tube alone) using electronic analytical balance (AND FR-200 MK II, Japan) and the tube containing WB clot was appropriately labeled.

In the early part of this study we have validated the unresected (uncut) and resected (cut) WB clots to ensure a uniform clot weight. The unresected clot showed more consistent results of DD trends when suspended in pool PPP for a period of time than the resected clot.

The quantitation of DD was done using STA Compact Coagulation Analyzer (STAGO) and was determined photometrically by the immunoturbidimetric method using Liatest kit (STAGO).

#### 2.1.3. Procedure for WB Clot Lysis Incubated in Pool PPP

This procedure was used as a control test when duration of incubation was assessed for fibrinolytic activity. To each of the tubes containing retracted WB clot, 1 mL of prepared pool PPP was added after thawing at 37°C. The tubes were covered by parafilm and incubated at 37°C for 3, 6, and 9 hrs. Four groups of tests were analyzed as follows: Group 1 as baseline (0 hour incubation), Group 2 incubated for 3 hrs, Group 3 for 6 hrs of incubation, and Group 4 for 9 hrs of incubation. Following incubation, the plasma was obtained after gentle shaking of the clot and was then removed by Pasteur pipette in microcentrifuge tube (bullet tube) and each glass tube containing clot was again weighed after pool PPP incubation the difference in weight before and after clot lysis was then subsequently recorded. The previously removed plasma containing RBCs and other particles due to WB clot lysis was spun at 1200 g for 5 min (Eppendorf 5424, USA) and then the supernatant was tested for the DD levels. These procedures were repeated 10 times to assess the DD and clot weight changes.

#### 2.1.4. Procedure for WB Clot Lysis Incubated in SK

Commercial lyophilized SK vial (15, 00,000 IU, CLS Behring GmbH, 35041 Marburg, Germany) was purchased as a powder. The powder was dissolved with 5 mL normal saline, as per the manufacturer's instruction. This solution was used as a stock from which suitable dilutions were made to study the fibrinolytic activity using *in vitro* WB clot performed in our laboratory. The stock solution was aliquot into small volumes in cryogenic vials and stored at −80°C until use. The solution was thawed at room temperature (~22°C) whenever needed and the unused portion was discarded. For the validation of the WB clot lysis procedure, four dilutions of thrombolytic drug (5, 30, 50, and 100 IU/mL) using SK were prepared using pool PPP as diluents with defined volume of 1 mL. WB clots were exposed to the SK in the above-mentioned dilutions. Following 1 hr incubation, the DD level and WB clot weight were measured as described above. The function of SK is to convert plasminogen to plasmin which in turn degrades the fibrin clot [15]. The normal range of plasminogen level in plasma is 0.75–1.60 *μ*/mL [16]. In the present study, we used PPP from apparently normal subjects who were expected to have normal plasminogen level. In pathological fibrinolysis such as disseminated intravascular coagulation (DIC), plasminogen level is usually low [16]. In this study, different concentrations of SK (mentioned above) were used and expected to have different fibrinolytic effects on the WB clot. A local intracoronary infusion of 20,000 IU by bolus maintained at 2000 units/minute was used in the treatment of myocardial infarction. The highest concentration, that is, 100 IU/mL, used in this study is 14.3 times more than the therapeutic dose for test tube equivalent (taking into consideration the fact that the WB clot is placed in a static milieu). Another reason for choosing these concentrations is because, at concentration more than 100 IU/mL, SK has a potent effect on the clot lysis difficult for assessment of DD linearity. The same procedure was performed for each concentration of SK and repeated 10 times to assess the DD and clot weight changes.

### 2.2. Confocal Microscopy Protocol and Staining Procedure

Confocal microscopic studies on WB clot have been reported and previously described in a few studies [2, 13, 14]. The reagents used in this study are fibrinogen fluorescence dye (Alexa Fluor 488 human fibrinogen conjugates (F-13191) purchased from Molecular Probes and prepared as per manufacturer's instructions. Stock solution was prepared by dissolving 5 mg of fibrinogen in 3.3 mL of 0.1 M sodium bicarbonate (NaHCO_3_) (PH 8.3) at room temperature. The working solution was prepared by adding 100 *μ*L fibrinogen (stock) dye to 6 mL distilled water. The complete solubilization was done with occasional gentle mixing for one hour. Stock solution was stored at 4°C for further use. The working solution (0.3 M) of merocyanine 540 fluorescent (MC-540) 25 mg/(MW = 569.67) (erythrocyte dye) was prepared by dissolving 3.4 mg in 20 mL distilled water. Commercially available phosphate buffered saline (PBS) was used as the main washing buffer in this study. The other buffer 0.1 M sodium bicarbonate (NaHCO_3_) was used as diluents for fibrinogen fluorescence dye.

Venous blood was collected strictly from O blood group volunteers and 180 *μ*L of WB was put in each well of 8-chambers polystyrene vessel tissue culture treated glass slide (BD falcon cultures, USA) according to the ratio used for WB clot lysis in the method described above. The WB clot was incubated at 37°C for 3 hrs to ensure complete clot retraction (as above). The serum was completely and gently removed from the chambers after 3 hrs. After that 120 *μ*L of the pool PPP (Group 1), stock solution of SK (Group 2) and SK + plasma, that is Group 3, at different concentrations (5, 30, 50, and 100 IU/mL) were added to the retracted WB clots which were formed in each well of the chambers. The labeling was done according to the period of incubation in pool PPP: 0, 3, 6, and 9 hrs at 37°C. On the other hand the labeling was done according to the SK concentration and incubated for one hour. The plasma was removed after each incubation period of time. The WB clot was washed by PBS three times for 3–5 min for each wash. The fibrin fibres were labeled with 600 *μ*L working concentration of the primary antibody (Alexa Fluor 488 human fibrinogen conjugate F-13191, Molecular Probes 5 mg, Invitrogen Life Technologies) which was freshly prepared using sodium bicarbonate buffer (PH 8.3) and it was incubated and added to the clot at room temperature for 15 min in dark area to avoid direct light. The stain was removed and the clot was washed 3 times using PBS, after which it was incubated with buffer for 5 min. Following that the buffer was then removed. Subsequently, 600 *μ*L of the merocyanine-labeled RBC-emission wavelength 520 nm (Molecular Probe USA) was added to the WB clot as RBC staining and incubated at room temperature for 45 min in dark room. After that the dye was washed using PBS 3 times for 3–5 min for each washing. The antifade fluorescence mounting medium was added before being examined by confocal microscopy. Images for fibrin and RBCs for each group were obtained on a Pascal 5 Axiovert inverted laser confocal microscope with a 63X lens using an argon and HeNe laser (Carl Zeiss, Germany). For confocal image of retracted WB clot, Alexa 488 labeled fibrin fibers in green, while the merocyanine 540 fluorescent dye (MC-540) labeled the RBC membranes in red colour.

### 2.3. Statistical Analysis

Statistical analyses were performed using PASW Statistics 19 (SPSS, Chicago, IL). Data were expressed as mean difference of DD within group for WB clot lysis, when incubated with plasma at different time and SK at different concentrations. The relationship between them was investigated by using repeated measures ANOVA within group analysis followed by pairwise comparison and one way ANOVA test followed by posthoc comparison, respectively. The WB clot weight between before and after plasma and SK incubation was compared using Wilcoxon Signed Rank test. A *P* value ≤0.05 was considered to be statistically significant.

## 3. Results

**Figure 1:**
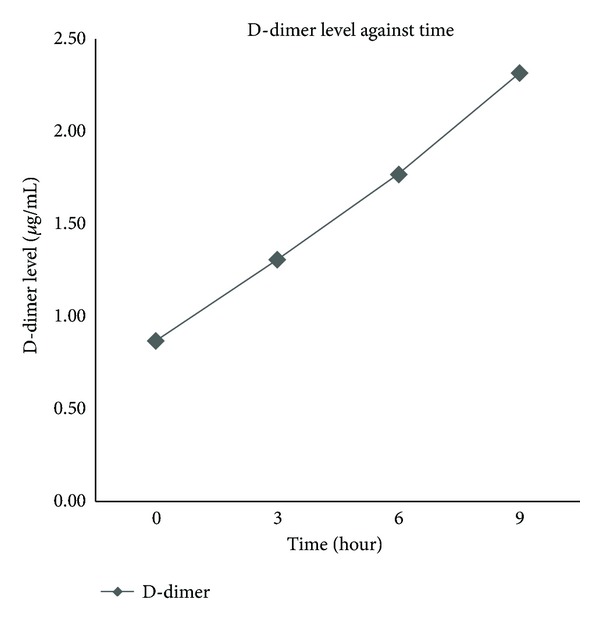
Line graph of D-dimer level against time of whole blood clot lysis in plasma.

There was a significant mean difference of DD within the group of unresected WB clot lysis in pool PPP (without SK) when compared between 0 and 3 hrs, 0 and 6 hrs, 0 and 9 hrs, 3 and 6 hrs, 3 and 9 hrs, and lastly 6 and 9 hrs (*P* values <0.007). These findings reflect a proportional increase in DD according to the incubation time. The mean DD (SD) according to time recorded at 3, 6, and 9 hrs is 1.30 (0.26), 1.77 (0.48), and 2.31 (0.37) *μ*g/mL, respectively (Figure 1).

**Figure 2:**
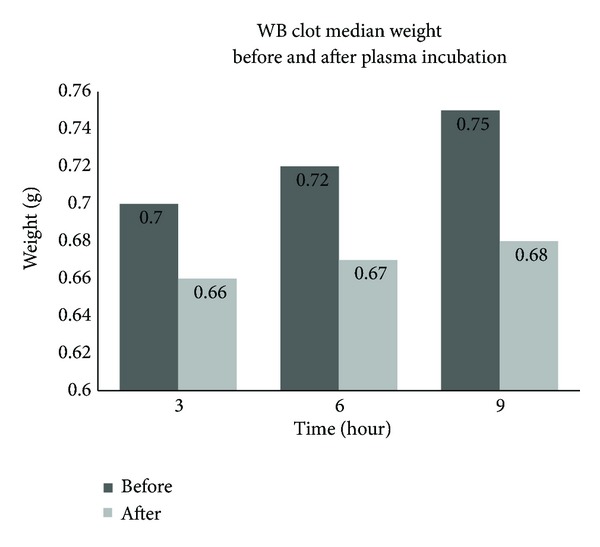
The difference in clot median weight between before and after pool PPP incubation.

There was a significant difference of median clot weight of unresected clot between before and after PPP incubation at 3, 6 and 9 hrs. The median (interquartile range) (IQR) weight before versus after incubation at 3 hrs was 0.7 (0.06) versus 0.66 (0.04); at 6 hrs 0.72 (0.06) versus 0.67 (0.07); at 9 hrs 0.75 (0.07) versus 0.68 (0.06) gram, respectively (*P* values <0.001). These findings reflect WB clot lysis activity before and after incubation in PPP in their respective groups (Figure 2). However there were no significant changes of clot weight after incubation between different times (0.66–0.68 g) as depicted in Figure 2.

**Figure 3:**
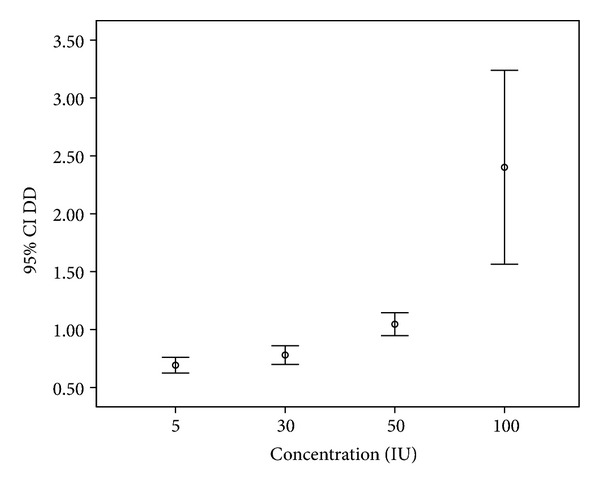
D-dimer level (*μ*g/mL) against various concentrations (IU/mL) of streptokinase using Box-and-Whisker plot.

The results for WB clot incubated in SK at different concentrations are demonstrated in Figure 3. The baseline level of DD from pool PPP for SK dilution was <0.50 *μ*g/mL. This reflects a proportional increase in DD level according to the SK concentrations. There was significant mean difference of DD between all concentrations except for concentration of 5 versus 30 IU/mL. The mean DD (SD) according to the SK concentrations for 5, 30, 50, and 100 IU/mL was 0.69 (0.12), 0.78 (0.14), 1.04 (0.14), and 2.40 (1.09) *μ*g/mL, respectively.

**Figure 4:**
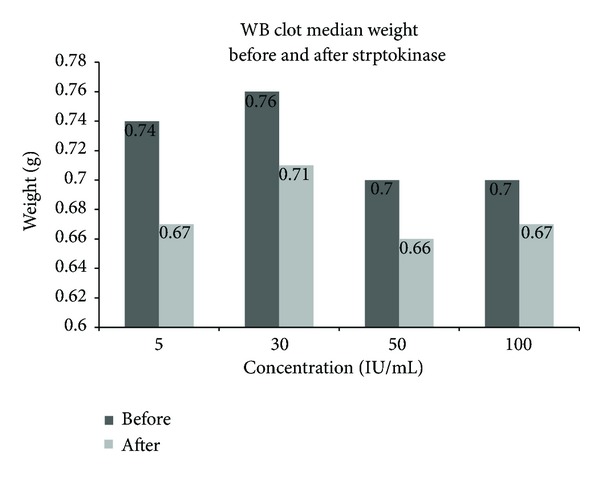
The difference in clot median weight between before and after SK induced fibrinolysis.

There was also a significant difference in median clot weight between before and after SK for each concentration: 5, 30, 50, and 100 IU/mL (*P* values <0.01). The median (IQR) weight before versus after SK incubation for each concentration at 5 IU/mL was 0.74 (0.08) versus 0.67 (0.06), at 30 IU/mL 0.76 (0.10) versus 0.71 (0.8), at 50 IU/mL 0.70 (0.10) versus 0.66 (0.12), and at 100 IU/mL 0.70 (0.09) versus 0.67 (0.10) gram, respectively (Figure 4).

While there was significant difference between before and after incubation at individual SK concentration, the median clot weight showed little or no difference between 5, 50 and 100 IU/mL post SK incubation. This indicates that the clot weight changes were not significant between the SK concentrations designed for this study.

### 3.1. Confocal Microscopy

**Figure 5:**
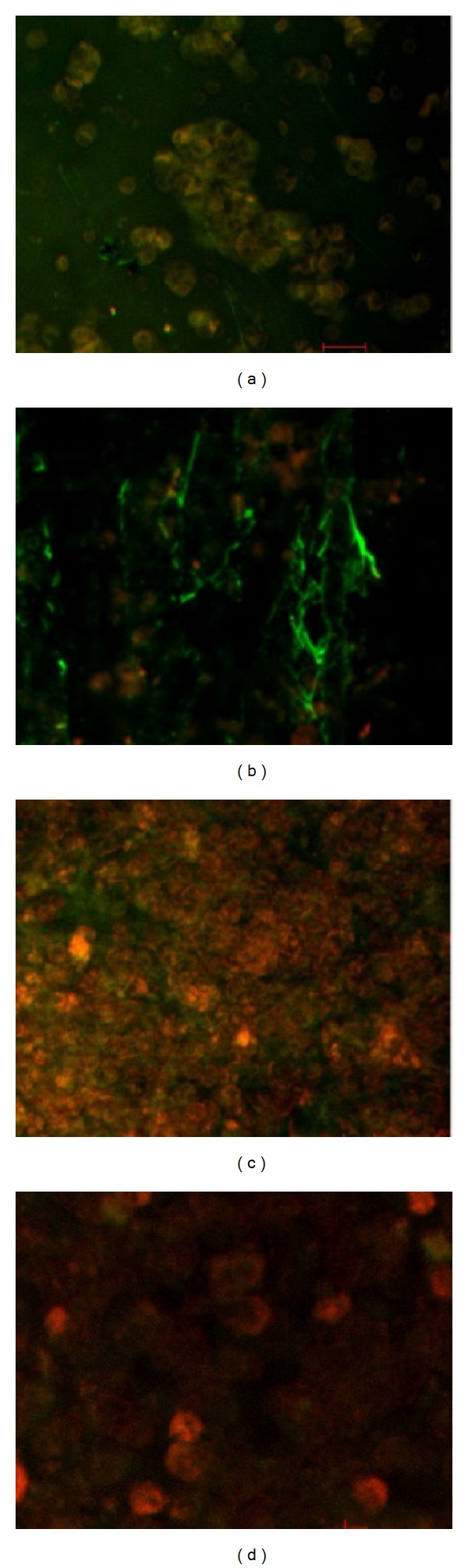
Confocal images for retracted WB clot incubated in pool PPP. (a) showed confocal image of normal retracted WB clot. (b), (c), and (d) showed retracted WB clot after 3, 6, and 9 hrs incubation in pool PPP, respectively. (a) shows confocal images of normal retracted WB clot showing fibrin mass surrounded and covered by RBCs (control untreated). (b) demonstrates a clear separation of fibrin from the RBCs after 3 hrs of WB clot incubation in PPP. (c) shows fibrin separation from the RBCs and a gradual loss of fibrin fibres after 6 hrs of incubation. (d) shows at 9 hrs of incubation in PPP minimal fibrin fibres with some remaining RBCs.

**Figure 6:**
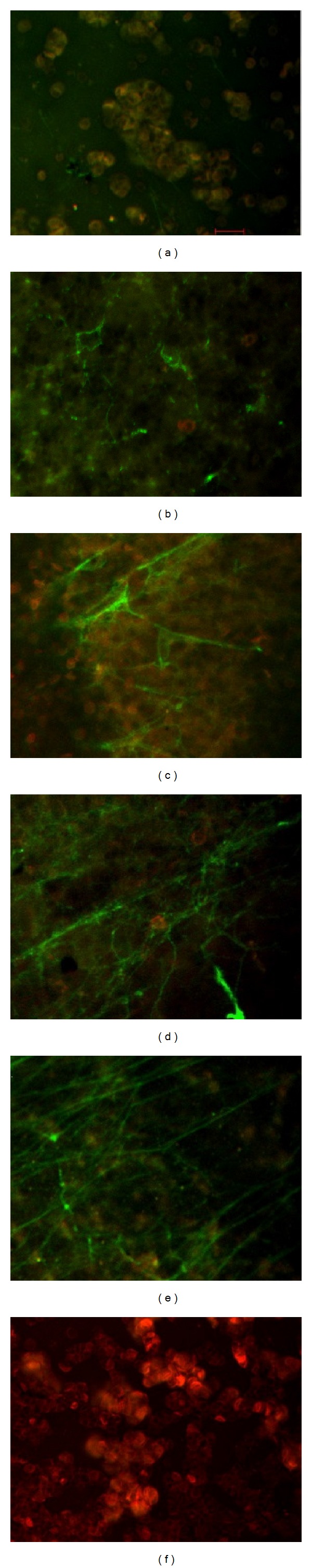
Confocal images for retracted WB clot treated with SK. (a) showed image of normal retracted WB clot. (b), (c), (d), (e) and (f) showed retracted WB clot treated with 5 IU/mL, 30 IU/mL, 50 IU/mL, 100 IU/mL, and 1500,000 IU (stock) of SK concentrations after 1-hour incubation, respectively. (a) demonstrates fibrin integrated with RBCs in retracted WB clot (control untreated). (b) shows 5 IU/mL concentration of SK where the fibrin started to separate from the RBCs. (c) depicts 30 IU/mL concentration of SK showing the fibrin that separated from the RBCs and a gradual thinning of fibrin fibres. (d) shows 50 IU/mL concentration of SK demonstrating fibrin separation from the RBCs and the fibrin fibres became much thinner and increased branching than 30 IU/mL. (e) shows 100 IU/mL concentration of SK where the fibrin almost separated from the RBCs and fibrin fibres became much thinner and increased branching than the 50 IU/mL SK. (f) shows 1500,000 IU (stock) of SK showing complete disappearance of fibrin from the RBCs.

Parallel experiments were performed *in vitro* in the presence of retracted WB clot incubated in pool PPP and SK. There was a gradual loss of fibrin fibres in WB clot according to the incubation time for pool PPP and concentrations of SK as shown in Figures 5 and 6, respectively.

## 4. Discussion

In this study combination of D-dimer, WB clot morphology, and WB clot weight were used for the validation of *in vitro* WB clot lysis method. The present method was modified from previously published articles and validated independently using the above-mentioned parameters [6, 17, 18]. The plasma was used as a medium to immerse the WB clot for the purpose of monitoring the coagulation parameters or products such as DD. The retracted WB clot when suspended in a plasma milieu became more sensitive to lysis with fibrinolytic agents than in buffer reagent. The plasma proteins such as plasminogen and fibrin that were entrapped in the clots might have contributed to their sensitivity to lysis; in addition, the plasma milieu contains soluble proteins such as plasminogen, tissue plasminogen activators, and *α*_2_ antiplasmin all of which also play crucial role in clot lysis [9]. The O blood group donors were used to ensure compatibility because the WB clot was created from O blood group individuals (however, plasma from A, B, and AB blood groups could also be used as sources of plasma because they do not usually produce haemolysis as a result of minor ABO incompatibility).

The present study used retracted WB clot as fresh WB clot was found not suitable for fibrinolytic studies [9]. The quantity of unbound plasminogen inside the clot (plasminogen/fibrin ratio) is higher in nonretracted clots than in retracted ones. This high ratio (which is as a result of high number of plasminogen molecules in close proximity to fibrin that can bind to partially degraded fibrin) makes the non retracted clots more prone to lysis with any type of activator. The ratio is even lesser in retracted clots as the pool of plasminogen is mainly located at the external surface of the clot [10]. Retracted WB clot increases the concentration of fibrin network and bound fibrinolytic inductors. Also fibrin induces the conversion of plasminogen to plasmin by plasminogen activators. In addition, the fibrin formed due to the clot retraction may stimulate the lysis activity [11]. Thrombolytic agents such as rt-PA cleave the fibrinogen and fibrin of the thrombus into fragments D and E, and these products are represented by the concentration of DD as a specific fibrin degradation product. Determination of DD concentration is a valid measure for quantifying the efficacy of fibrinolytic therapy [19].

In this method validation, retracted WB clot lysis in PPP was used as control test in which the lysis was allowed to occur spontaneously with time. The rate and concentrations of DD were lower than the treated clot using SK. This finding is supported by previous studies, which used spontaneous clot lysis activity in buffer or plasma as control [11, 12]. Our study showed that DD levels increased with time of incubation in plasma and with increasing concentrations of SK which were also statistically significant (*P* < 0.05). A comparable finding with the plasma clot has been reported previously [19]. This study found that DD provides more discriminative results for assessment of *in vitro* whole blood clot lysis activity than clot weight. Increasing trend of DD but not the clot weight was observed when the clot was allowed to undergo natural lysis with time. This finding suggests that DD is a suitable and sensitive marker for assessment of *in vitro *WB clot lysis activity. The increasing trend of DD agrees with the previous report that used haemoglobin and radioactive method to evaluate the clot lysis activity [11, 12].

As mentioned earlier in the methods, there were differences between resected and unresected WB clot. There was a problem in standardizing the clot weight. Although the same blood volume was used from healthy donors with normal haemoglobin level, it was observed that, the clot weight after retraction varied from one tube to another. It was difficult to get an acceptable uniform weight unless the clots were resected so as to fix their weight. On the bases of this, the value of clot weight was fixed in the preliminary study by resecting the clots to standardize their weight. It was suggested that by using resected WB clot, fibrinolytic activity might be affected probably as a result of exposing the inner layer of the clot to PPP. This in turn leads to inconsistencies in the results of the resected clot compared with the unresected one. Previous studies have reported clot weight in the form of ranges for *in vitro* studies [17, 18].

In the present study we included WB clot weight as previous studies had used it as indicator of clot lysis activity. The median weights the whole blood clot after PPP incubation and with SK were significantly lower than before PPP and SK incubation. This result correlates with the previous studies [6, 18]. However we observed that, at different concentrations of SK (5, 50, and 100 IU/mL), there was not much difference in weight changes. This could probably imply that the SK doses used here were inappropriate or the lytic activity has reached its plateau despite of the different concentrations used. This effect is probably due to a limiting factor from natural inducers of fibrinolysis such as plasminogen which control the clot to continue the lytic process. However, the concentrations of DD increased significantly with SK in these concentrations (5, 50, and 100 IU/mL). This finding also explained the sensitivity of the two different methods in detecting fibrinolytic activity particularly at molecular level.

The branching of fibrin fibres was found to be larger in size, thinner, and separated from RBC with the higher levels of DD. The size of the fibrin branching increased and then disappeared with the increasing concentration of SK. In PPP, fibrin was separated from RBC and disappeared with time. A study has shown increased porosity of fibrin when treated with thrombin and factor Xa inhibitors using 3D confocal microscopy [20]. This study applied qualitative assessment of the blood clot morphology and hence the finding was not reported in quantitative measurement (such as grading system). In future, evaluation of the WB clot morphology subjected to fibrinolysis could be improved by applying appropriate quantitative measurements for more objective results.

The effect of SK on fibrin imaging was evaluated by optimization of the untreated fibrin as shown by the representative image in Figures 5(a) and 6(a). It was found that fibrin of WB clot incubated in PPP at 3 and 6 hrs showed highly branching feature and loss of fibres at 9 hrs. The WB clot on confocal images also demonstrated that the fibrin fibres were thicker in PPP than in the SK treated clot. WB clot underwent gradual loss of fibrin fibres when incubated in PPP according to the incubation time and there was minimal or no fibrin fibres at 9 hrs.

In contrast, there was a gradual loss of fibrin fibres in WB clot treated in SK at different concentrations: 5, 30, 50, 100 IU/mL, and 1,500,000 IU (stock solution). There was also increasing branching and loss of scaffolding features (red cells integrated in the fibrin mass) at 100 IU/mL and eventually the fibres disappeared with higher concentration of SK such as at 250, 1000 IU/mL and stock solution.

Previous study indicated that the fibres ends are rarely seen in an undamaged normal fibrin clot. This is because the protofibrils lateral aggregation produces clots with thick fibres and few branch points. However, the inhibition of lateral aggregation due to fibrinolytic process tends to produce clots with thin fibres and numerous branching points which is in agreement with our findings where increased branching is seen at 30 IU/mL compared to 5 IU/mL [1].

In this study the fibrin fibres were thinner in SK incubation than with the control test (incubated in PPP) at 5 IU/mL and 30 IU/mL versus 3 hrs of incubation in PPP. This shows the effect of penetration of SK into the WB clot. WB clot incubated in PPP undergoes natural fibrinolysis induced by plasma proteins. In these two methods, the structure of the fibrin networks underwent significant changes in architecture during the lytic process. The fibrin fibres became thinner and, underwent gradual loss based on the increasing concentrations of SK and time of incubation in PPP [13, 14, 20]. While optimizing SK concentrations for the method, we found that 100 IU/mL was more suitable for *in vitro* method. The highest concentration (100 IU/mL) used in this study is 14.3 times more than the therapeutic dose for test tube equivalent. There are limitations in this study; for example, the concentration of streptokinase used was probably low and difficult to optimize with the size of the blood clot. In addition the time for incubation with plasma containing streptokinase was short (1 hour). Although increased levels of D-dimer could be a marker of initiation of biochemical reactions involved in fibrinolysis after incubating clots with plasma containing streptokinase, under conditions used in this experiment one would not expect significant reduction of blood clot weight regardless of concentration of streptokinase used. In future studies, it would be interesting to compare D-dimer levels after same period of time (e.g., 1 hour or longer) in clots exposed only to plasma on one hand and in clots exposed to plasma and streptokinase on the other hand.

## 5. Conclusion

The modified *in vitro *WB clot lysis method has been evaluated using combination of DD and confocal microscopy. Unresected retracted WB clot was found suitable for this method validation. DD is more appropriate than clot weight in discriminating the fibrinolytic activity. This WB clot method is useful for investigation of factors affecting the WB clot lysis or for exploring the potential fibrinolytic agents in a clinical laboratory as a preliminary research work.
